# Trends of regenerative tissue engineering for oral and maxillofacial reconstruction in veterinary medicine

**DOI:** 10.3389/fvets.2024.1325559

**Published:** 2024-02-21

**Authors:** Steven Dwi Purbantoro, Teeanutree Taephatthanasagon, Medania Purwaningrum, Thanyathorn Hirankanokchot, Santiago Peralta, Nadine Fiani, Chenphop Sawangmake, Sirirat Rattanapuchpong

**Affiliations:** ^1^Veterinary Stem Cell and Bioengineering Innovation Center (VSCBIC), Faculty of Veterinary Science, Chulalongkorn University, Bangkok, Thailand; ^2^Veterinary Stem Cell and Bioengineering Research Unit, Faculty of Veterinary Science, Chulalongkorn University, Bangkok, Thailand; ^3^Department of Biochemistry, Faculty of Veterinary Medicine, Universitas Gadjah Mada, Yogyakarta, Indonesia; ^4^Department of Clinical Sciences, College of Veterinary Medicine, Cornell University, Ithaca, NY, United States; ^5^Department of Pharmacology, Faculty of Veterinary Science, Chulalongkorn University, Bangkok, Thailand; ^6^Center of Excellence in Regenerative Dentistry, Faculty of Dentistry, Chulalongkorn University, Bangkok, Thailand; ^7^Academic Affairs, Faculty of Veterinary Science, Chulalongkorn University, Bangkok, Thailand

**Keywords:** oral and maxillofacial defects, oral and maxillofacial reconstruction, tissue engineering, maxillofacial regeneration, veterinary

## Abstract

Oral and maxillofacial (OMF) defects are not limited to humans and are often encountered in other species. Reconstructing significant tissue defects requires an excellent strategy for efficient and cost-effective treatment. In this regard, tissue engineering comprising stem cells, scaffolds, and signaling molecules is emerging as an innovative approach to treating OMF defects in veterinary patients. This review presents a comprehensive overview of OMF defects and tissue engineering principles to establish proper treatment and achieve both hard and soft tissue regeneration in veterinary practice. Moreover, bench-to-bedside future opportunities and challenges of tissue engineering usage are also addressed in this literature review.

## 1 Introduction

**Figure 1:**
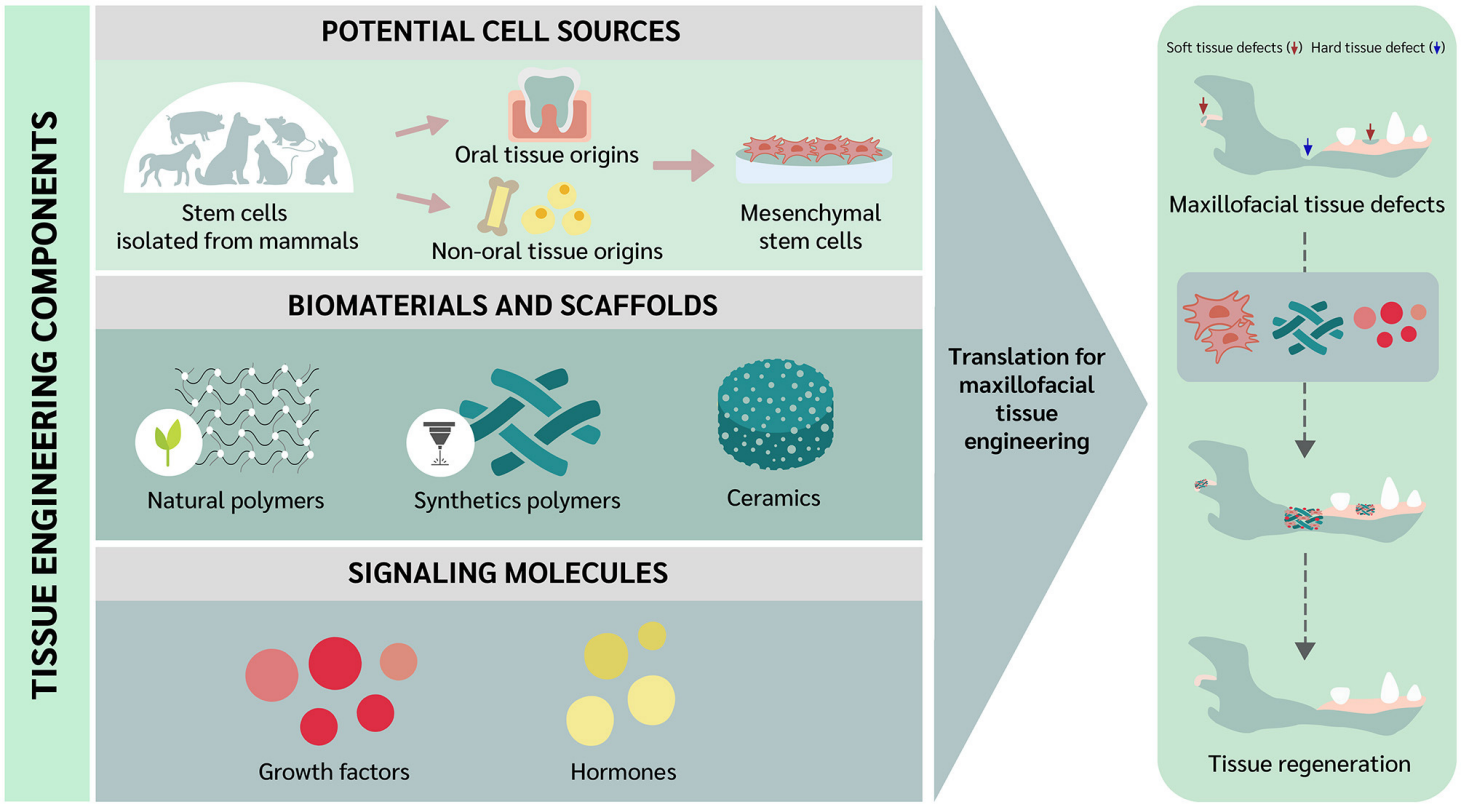
Translational concept of oral and maxillofacial tissue engineering in veterinary medicine.

As is the case with other clinical disciplines, oral and maxillofacial (OMF) surgery is constantly evolving, always aiming to improve treatment capability. Veterinary OMF surgeons often encounter technical challenges when reconstructing hard and soft tissue defects, and alternative approaches that can help minimize some of the limitations are necessary (1). In this context, tissue engineering was introduced as a novel approach to tissue regeneration and repair (2), and extensive studies have been conducted aiming to overcome the drawbacks of conventional solutions in OMF surgery. In addition, tissue regeneration implies the involvement of tissue component substitution to the damaged tissue returning to the normal state. While tissue repair involves a “*patching*” mechanism, with connective tissue deposition such as a scar to provide enough integrity to the injured tissue. However, excessive connective tissue deposition, known as fibrosis, in some tissues may alter the tissue functions due to the inability of tissue remodeling (3–5). As noted by Langer and Vacanti, tissue engineering comprises the use of stem cells, scaffolds, and/or signaling molecules (2). Interestingly, trend of using differentiated or somatic cells can be employed for tissue engineering as well (6). Recently, the trend of scaffold-free approaches have been raised and elaborated in the tissue engineering field. The approaches focus on the overcoming the issues of cell survival and localization, immune reaction, and ECM protection and growth factor storage (7, 8). Among the different types of cells, stem cells have been highlighted because primary cells have limitations in cell resource and risk for disease transmission (9). Stem cells to be used for tissue engineering may be embryonic stem cells (ESCs), adult stem cells such as mesenchymal stem cells (MSCs) and hematopoietic stem cells (HSCs), or induced pluripotent stem cells (iPSCs) (9). However, MSCs have received much attention due to their potential for clinical use in both humans and animals (10). Mesenchymal stem cells can be divided based on the collection site into oral and non-oral tissue origins (11). Scaffolds can be utilized as a carrier for cells and signaling molecules (9). In this review, we elaborate on some of the fundamental concepts underlying tissue engineering and describe current trends directed toward future applications in veterinary OMF surgery. The tissue engineering concept to be translated for OMF regeneration in veterinary practice is illustrated in Figure 1.

The OMF region is composed of hard and soft tissues. Hard tissues include the teeth and multiple bones, such as the maxillary, incisive, mandibular, nasal, frontal, temporal, zygomatic, and palatine bones, among many others. The mandible articulates with maxillary structures via the temporomandibular joint (TMJ). Associated soft tissues include the TMJ disc and attachment, cartilage and attachment, tongue, oral mucosal surfaces, lymphoid tissue, salivary glands, muscles, nerves, vascular system, and skin (12). Understanding the complex anatomy and functionality of the OMF region is essential when diagnosing associated disorders and implementing proper treatments that can achieve an optimal outcome.

Anatomical defects that affect the functionality of the OMF region are common in both humans and animals. Generally, OMF defects can be classified as congenital or acquired (13). Orofacial clefts represent a relatively prevalent example of a congenital OMF defect of high clinical relevance that requires surgical reconstruction. Conversely, acquired defects are a frequent sequela of oncological surgery and traumatic injuries of the OMF region. Several classifications or grading schemes have been reported in humans based on the specific anatomical location of the OMF defect. Although there is no standardized classification system for animals, OMF traumatic injuries (e.g., vehicular accident, animal bites, blunt force, and others) and congenital defects (clefts of the lip, palate, or both) have been comprehensively documented in dogs (14–17).

## 2 Tissue engineering for OMF reconstruction

Depending on the nature, extent, location, and stage of the OMF disorder, conventional surgical techniques may be contraindicated due to the inability to reconstruct existing or resultant defects (18). Moreover, allogenic organ transplantation and prosthetic rejection due to an adverse immune response may pose an important obstacle (19). Tissue engineering is an interdisciplinary field that combines biology and engineering principles to establish functional substitutes for damaged tissue (2) with the aim of addressing the aforementioned limitations, especially for the application in OMF reconstruction.

Using the current state of the art tissue engineering solutions, current conventional surgical techniques, such as autologous bone graft surgery could be complemented or replaced to address the general surgical concerns of donor site morbidity, poor anatomical match, insufficient graft volume, graft resorption, and rejection (20, 21). To reconstruct orofacial clefts in dogs, soft tissue flaps are typically harvested from the region adjacent to the defect. In severe cases where the cleft is too wide for local tissue flaps, tooth extraction and a staged approach has been described (22). Surgical site morbidity associated with soft tissue harvest sites, tooth extraction, as well as dehiscence rate (23), are the primary complications of this concern. Regarding acquired bone defects that result in functional disturbances, treatments have been variable and span the spectrum of surgical and non-surgical options (24, 25). None of the techniques described are without complication. Alternatively, tissue regeneration is an option that aims to restore and replace damaged tissue that overcomes conventional correction techniques by applying tissue engineering field (26).

## 3 Potential cell sources

As previously mentioned, stem cells represent the main cell source used for tissue regeneration because of their self-renewal, multilineage differentiation and high proliferative potential (19, 26, 27). Mesenchymal stem cells (MSCs) are the primary focus of this review given that they can differentiate into hard and soft tissue cells and be tissue-engineered for repair of different OMF defects. With this in mind, we further divide cell sources according to origin as either oral or non-oral MSCs. To be noted, craniomaxillofacial tissues have not only oral MSCs, but also others including suture-derived MSCs and periosteum-derived stem cells (28, 29). However, this literature review focuses on the oral/mouth-derived MSCs because suture- and periosteum-derived MSCs are still sparsely reported.

### 3.1 Oral tissue origins

Oral derived human stem cells have been previously reported (11, 28, 29). In 2000, dental pulp stem cells (DPSCs) were first isolated from permanent third molar teeth and their stemness properties were demonstrated (30). Successful isolation of DPSCs was followed by stem cells from exfoliated deciduous teeth in 2003 (31), periodontal ligament stem cells (PDLSCs) in 2004 (32), dental follicle stem cells (DFSCs) in 2005 (33), stem cells from apical papilla (SCAP) in 2006 (34), and gingival MSCs (GMSCs) in 2010 (35). It has been reported that oral stem cells are advantageous in terms of collection due to its easiness with least invasive way, convenience, and affordable, cryopreservable, and transportable. Additionally, the cells are able to have interaction with scaffold and signaling molecule (36–38). These previous studies showed that the cells exhibited the expected behavior and potentiality such as high proliferation rate and multilineage differentiation ability. According to the International Society for Cellular Therapy (ISCT), multilineage differentiation ability is that MSCs can be differentiated into osteoblasts, adipocytes, and chondroblasts *in vitro* (39). Many studies also reported confirmational expression of pluripotency regulators and proliferative marker either as in genes or proteins, e.g., SRY-box transcription factor (TF) 2 (SOX2), REX1 TF (REX1), octamer-binding TF 4 (OCT4), and homeobox TF Nanog (NANOG), and proliferation marker Ki67 antigen (Ki67), respectively (40–42). In addition, this literature review presented the results of MSC surface markers both from flow cytometry and polymerase chain reaction (PCR) results, including CD146 (MSC multipotency), CD44 (cell-matrix interaction), CD29 (cell adhesion), CD73 (cell migration and anti-inflammatory property) CD90 (cell adhesion, migration, homing, proliferation, apoptosis, and differentiation), CD105 (cell migration, proliferation, and differentiation), and CD45 (hematopoietic stem cell (HSC) marker) (43–49).

Stem cells have also been isolated from the oral tissues of non-human mammalian species including canines, felines, equines, chimpanzees, swines, murines, and minipigs (34, 43, 50–53). Among these, canine-derived oral-origin MSCs have been widely studied (41, 43, 54–64). In contrast, feline-derived oral-origin MSCs have only been sparsely reported. Therefore, we have drawn parallels between canine and human oral-origin MSCs. Indeed, one study showed that canine DPSCs exhibited similar stemness properties to human DPSCs including adherence to a plastic surface and fibroblast-like morphology (43). The same study also found that canine DPSCs have a higher proliferation rate than human bone marrow-derived mesenchymal stem cells (BM-MSCs), yet lower than human DPSCs. In addition, human DPSCs have a higher number in colony formation than canine DPSCs (63). Moreover, canine DPSCs showed higher mRNA expression of *CD146* and *Nanog* (43). In addition, previous studies reported that canine DPSCs recapitulate the pluripotency of human DPSCs with the ability to differentiate into osteogenic, adipogenic, and chondrogenic lineages, but not neurogenic lineage (43, 57, 65–67). Apart from that, the osteogenic and adipogenic differentiation potential of canine DPSCs was weaker compared to human DPSCs (43, 63). The *RUNX2* expression of human and canine DPSCs on day 7 after osteogenic induction showed ~2.5 and 0.6 times comparing to control group (non-induced group), respectively. For adipogenic differentiation, canine DPSCs showed that fewer formation of intracellular lipid droplets compared to human DPSCs (43, 63). Interestingly, previous studies have shown that human DPSCs can differentiate into insulin-producing cells (IPCs) (68–70). Feline DPSCs have also exhibited MSC properties, with spindle-shaped cells that have the ability to form colonies (71). Like other MSCs, feline DPSCs were also able to differentiate into osteogenic, adipogenic, and chondrogenic. However, the MSC surface marker expression profile of feline DPSCs has not been established.

Similarly, stem cells from human exfoliated deciduous teeth (SHED) have been isolated. One study showed that SHEDs are fibroblast-like shaped multipotent stem cells with proliferative and clonogenic capacity (31). It was noted that the highest number of population doublings was from SHED, followed by human DPSCs and BM-MSCs. Just like other subgroups of MSCs, SHEDs were shown to express CD146 and STRO-1. Additionally, SHEDs can differentiate into odontogenic, osteogenic, adipogenic, and neurogenic lineages in a specific medium. Moreover, odontoblasts were found after SHED transplantation for 8 weeks into immunocompromized mice, being unable to completely regenerate dentin-pulp-like complex, like human DPSCs. Similarly, stem cells derived from canine exfoliated deciduous teeth are termed SCED. One study reported that SCED express CD105 and CD90 but not CD45 (59). Also, SCED were able to differentiate into neurogenic lineage (59). Unfortunately, a literature search did not yield any studies further characterizing SCED. In addition, stem cells from equine and ovine exfoliated deciduous teeth have been reported and showed fibroblastic-like morphology and proliferative property (72, 73). However, stem cells from exfoliated deciduous tooth from feline, porcine, bovine, caprine, non-human primate (NHP), rabbit, rat, and murine have not been reported.

In 1985, progenitor cells that reside in the periodontal ligament of mice were reported (74). The multipotency of PDLSCs was subsequently investigated. In humans, PDLSCs have typical fibroblast-like morphology, can form colonies, and can differentiate into osteoblasts, adipocytes, chondrocytes, and neurogenic (32, 75, 76). Consistent with MSCs, PDLSCs highly expressed CD73, CD90, CD105, and STRO-1 but negatively expressed CD45 (63). Furthermore, an *in vivo* study of human PDLSCs in combination with hydroxyapatite (HA)/tricalcium phosphate (TCP) showed their ability to differentiate into cementum-like, PDL-like structures and to generate collagen fibers in immunocompromized mice (32). In the canine model, canine PDLSCs exhibited similar morphology and MSCs marker to human PDLSCs. The ability to differentiate into osteogenic, adipogenic, chondrogenic, and neurogenic lineages was also observed in canine PDLSCs (63, 64). However, canine DPSCs have a weaker potential to differentiate into osteoblasts and adipocytes compared to human PDLSCs (64). In addition, canine PDLSCs were reported to have ~2 times lower colony formation than human PDLSCs (63). Otherwise, canine cells combined with HA could still generate cementum-like, PDL-like structures and collagen fibers, similar to human cells (64).

A report of human DFSCs revealed a fibroblast-like morphology. Cells were proliferative and formed colonies. Like with other MSCs, human DFSCs could be induced into osteogenic, adipogenic, and neurogenic lineages (33, 77). Moreover, cells expressed CD29, CD44, CD90, CD105, and CD166, but no hematopoietic markers. In the same study, the authors isolated canine DFSCs and cultured them into cell sheets. However, there are no records regarding the characteristics of canine DFSCs.

Stem cells from apical papilla (SCAP) also exhibited MSC characteristics including fibroblast-like morphology and expression of STRO-1 as well as the other MSCs surface markers, and can differentiate into osteogenic and adipogenic lineages (34). A literature search did not yield any reports of SCAP isolation from canine teeth, however.

Given that GMSCs can be collected relatively easily, human and canine GMSCs were compared for their surface marker expression and osteogenic potential (56). Human GMSCs were found to express CD73, CD90, and CD105 and were able to differentiate into the osteogenic lineage. Conversely, canine GMSCs did not express CD73 or CD105, but did express CD90, and were able to differentiate into the osteogenic lineage. Unlike canine, human GMSCs have been characterized in several studies. One study revealed that human GMSCs are fibroblast-like shaped, and expressing several MSCs surface markers, such as CD44 and CD13, and have the capability to generate osteoblasts, adipocytes, and chondrocytes *in vitro* (78). Another study reported that human GMSCs proliferation was faster than BM-MSCs without additional growth factors, and cells were found to be clonogenic (79).

### 3.2 Non-oral tissue origins MSCs

Mesenchymal stem cells can be isolated from non-oral tissues, such as bone marrow (BM-MSCs) (61, 65), adipose (AD-MSCs) (61), umbilical cord (UC-MSCs), Wharton's jelly (WJ-MSCs), muscle, and liver. In this review, we focus on BM-MSCs and AD-MSCs.

Collection sites to aspirate BM-MSCs are either the iliac crest, the femoral shaft, or the proximal humerus, both in humans and canines (55, 80–82). As its harvesting protocol is invasive (81, 83), post-operative pain and risk of infection should be well-managed before the procedure is performed (84). Fibroblast-shaped morphology has been observed in human, canine, and feline BM-MSCs (61, 62, 65, 85). Canine BM-MSCs have been shown to express CD73, CD90, and CD146, but not CD45 (61, 62, 65). Humenik et al. demonstrated that canine BM-MSCs also express CD29 (86). Like canine BM-MSCs, human BM-MSCs were shown to express CD73, CD90, CD105, and CD146, but not CD45 (62). In the same study, human and canine BM-MSCs were also found to be clonogenic. Unlike canine and human BM-MSCs, feline BM-MSCs were only expressed CD9, CD44, and MHC1 as MSC surface markers (85). The mRNA expression of *SOX2, NANOG*, and *OCT4* as pluripotency genes was detected in both human and canine BM-MSCs (41, 87). Moreover, we have found that canine, feline, and human BM-MSCs are able to differentiate into many lineages, such as osteogenic, adipogenic, and chondrogenic (41, 55, 61, 65, 85, 88). In addition, both human and feline BM-MSCs are able to differentiate into neurogenic lineage (41, 85). A collective analysis mentioned that BM-MSCs across species have differences and uniqueness of stemness characteristics. To implement this result, dissemination of stemness characteristics is required for further clinical application. This collective analysis of BM-MSCs has been reported in the previous publication (81).

Previous studies have noted that human and canine AD-MSCs exhibit the same fibroblast-like morphology as BM-MSCs (61, 89). Human AD-MSCs can be isolated from neck or abdominal adipose tissues (89). In canines, adipose tissue can be obtained from subcutaneous, omental, and inguinal deposits and even biopsied fat (58, 61). Like BM-MSCs, canine AD-MSCs are able to express MSCs surface markers, such as CD90 and CD73, but not hematopoietic stem cells (HSCs) marker CD45 (61). Moreover, canine AD-MSCs are considered pluripotent based on protein expression of Oct4, Nanog, and Sox2 expressions (58). Like canine AD-MSCs, human AD-MSCs have been shown to express CD90, CD105, CD44, and CD73, but not CD45 (89). The same study found that human AD-MSCs expressed proteins such as Nanog, Sox2, and SSEA4, like canine AD-MSCs. In addition, human and canine AD-MSCs can differentiate into osteogenic, adipogenic, and chondrogenic lineages (58, 61, 89). Other studies have also reported that canine AD-MSCs have the ability to differentiate into IPCs (54, 61). It has been shown that AD-MSCs can also be isolated from felines (90, 91). These studies have noted that cell morphology was similar to other species and were proliferative, and that cells expressed CD9, CD44, CD90, and CD105 and could differentiate into osteogenic, adipogenic, and chondrogenic lineages (90, 91). To date, there is no data to provide collective analysis of AD-MSCs.

Of note, some studies include BM-MSCs and AD-MSCs as MSCs of oral-origin. Indeed, mandibles represent a potential collection site (29). Similarly, MSCs have been collected from neck fat in equines and the corresponding MSC characteristics and wound healing potential have been documented (92). A comprehensive summary of oral and non-oral potential cell sources from human, canine, feline, and equines is presented in Table 1 and livestock and laboratory animals are presented in Tables 2, 3, respectively.

**Table 1:** Potential cell source of oral and non-oral origin of human, canine, feline, and equine.

**Source**	**Species**	**Characteristics**												**Potential differentiation**					**References**
		**Fibro**	**Prolif**	**CD44**	**CD73**	**CD90**	**CD105**	**CD146**	**STRO-1**	***Nanog***	***Rex1***	***Oct4***	***Ki67***	**Osteo**	**Chondro**	**Adipo**	**Neuro**	**IPCs**	
DPSCs	Hu	+	+	+	+	+	+	+^G^	+	+	+	+	+	+	+	+	+	+	(43, 57, 66–70)
	Ca	+	+	N/A	+	+	+	+^G^	+	+	+	+	+	+	+	+	+	N/A	(43, 65)
	Fe	+	+	N/A	N/A	N/A	N/A	N/A	N/A	N/A	N/A	N/A	N/A	+	+	+	N/A	N/A	(71)
	Eq	+	+	+	N/A	+	+	N/A	N/A	N/A	N/A	N/A	N/A	+	+	+	N/A	N/A	(93)
SED	Hu	+	+	N/A	N/A	N/A	N/A	+	+	N/A	N/A	N/A	N/A	+	+	+	N/A	N/A	(31)
	Ca	N/A	N/A	N/A	N/A	+	N/A	N/A	N/A	N/A	N/A	N/A	N/A	N/A	N/A	N/A	+	N/A	(59)
	Fe	N/A	N/A	N/A	N/A	N/A	N/A	N/A	N/A	N/A	N/A	N/A	N/A	N/A	N/A	N/A	N/A	N/A	N/A
	Eq (fo)	+	+	N/A	N/A	N/A	N/A	N/A	N/A	N/A	N/A	N/A	N/A	N/A	N/A	N/A	N/A	N/A	(72)
PDLSCs	Hu	+	+	+	+	+	+	N/A	+	+	+	+	+	+	+	+	+	N/A	(32, 60, 63, 75, 76, 94)
	Ca	+	+	N/A	+	+	+	+	+	N/A	N/A	N/A	N/A	+	+	+	+	N/A	(63, 64)
	Fe	N/A	N/A	N/A	N/A	N/A	N/A	N/A	N/A	N/A	N/A	N/A	N/A	N/A	N/A	N/A	N/A	N/A	N/A
	Eq	+	+	N/A	N/A	N/A	N/A	N/A	N/A	N/A	N/A	N/A	+	+	+	+	N/A	N/A	(53, 95–97)
DFSCs	Hu	+	+	+	N/A	+	+	N/A	N/A	N/A	N/A	N/A	N/A	+	N/A	+	+	N/A	(33, 77)
	Ca	N/A	N/A	N/A	N/A	N/A	N/A	N/A	N/A	N/A	N/A	N/A	N/A	N/A	N/A	N/A	N/A	N/A	N/A
	Fe	N/A	N/A	N/A	N/A	N/A	N/A	N/A	N/A	N/A	N/A	N/A	N/A	N/A	N/A	N/A	N/A	N/A	N/A
	Eq	N/A	N/A	N/A	N/A	N/A	N/A	N/A	N/A	N/A	N/A	N/A	N/A	N/A	N/A	N/A	N/A	N/A	N/A
SCAP	Hu	+	N/A	N/A	+	+	+	+	+	N/A	N/A	N/A	N/A	+	N/A	+	N/A	N/A	(34)
	Ca	N/A	N/A	N/A	N/A	N/A	N/A	N/A	N/A	N/A	N/A	N/A	N/A	N/A	N/A	N/A	N/A	N/A	N/A
	Fe	N/A	N/A	N/A	N/A	N/A	N/A	N/A	N/A	N/A	N/A	N/A	N/A	N/A	N/A	N/A	N/A	N/A	N/A
	Eq	N/A	N/A	N/A	N/A	N/A	N/A	N/A	N/A	N/A	N/A	N/A	N/A	N/A	N/A	N/A	N/A	N/A	N/A
GMSCs	Hu	+	+	+	+	+	+	N/A	N/A	N/A	N/A	N/A	N/A	+	+	+	N/A	N/A	(56, 78, 79)
	Ca	N/A	N/A	N/A	N/A	+	N/A	N/A	N/A	N/A	N/A	N/A	N/A	+	N/A	N/A	N/A	N/A	(56)
	Fe	N/A	N/A	N/A	N/A	N/A	N/A	N/A	N/A	N/A	N/A	N/A	N/A	N/A	N/A	N/A	N/A	N/A	N/A
	Eq	+	+	N/A	N/A	N/A	N/A	N/A	N/A	N/A	N/A	N/A	N/A	+	+	+	N/A	N/A	(53)
BM-MSCs	Hu	+	N/A	N/A	+	+	+	+	N/A	+	N/A	+	N/A	+	+	+	+	N/A	(41, 55, 62, 81)
	Ca	+	+	N/A	+	+	N/A	+	N/A	+	+	+	+	+	+	+	+	+	(61, 65, 81, 86, 87)
	Fe	+	+	+	N/A	N/A	N/A	N/A	N/A	N/A	N/A	N/A	N/A	+	+	+	+	N/A	(81, 85, 88)
	Eq	+	+	N/A	N/A	N/A	N/A	N/A	N/A	+	N/A	+	N/A	+	+	+	N/A	N/A	(98–100)
AD-MSCs	Hu	+	N/A	+	+	+	+	N/A	N/A	+	N/A	N/A	N/A	+	+	+	N/A	N/A	(89)
	Ca	+	+	+	+	+	N/A	N/A	N/A	+	+	+	+	+	+	+	N/A	+	(54, 58, 61)
	Fe	+	+	+	N/A	+	+	N/A	N/A	N/A	N/A	N/A	N/A	+	+	+	N/A	N/A	(90, 91)
	Eq	+	+	+	N/A	+	+	N/A	N/A	N/A	+	N/A	N/A	+	+	+	N/A	N/A	(101–103)

DPSCs, dental pulp stem cells; SED, stem cells from exfoliated deciduous teeth; PDLSCs, periodontal ligament stem cells; DFSCs, dental follicle stem cells; SCAP, stem cells from apical papilla; GMSCs, gingival mesenchymal stem cells; BM-MSCs, bone marrow-derived mesenchymal stem cells; AD-MSCs, adipose-derived mesenchymal stem cells; Hu, human; Ca, canine; Fe, feline; Eq, equine; fo, foal; Fibro, fibroblast-like shaped; Prolif, Proliferative; Osteo, Osteogenic; Chondro, Chondrogenic; Adipo, Adipogenic; Neuro, neurogenic; IPCs, insulin-producing cells; +, positively expressed by proteins; +^G^, positively expressed by gene; N/A, not available.

**Table 2:** Potential cell source of oral and non-oral origin of livestock animals.

**Source**	**Species**	**Characteristics**												**Potential differentiation**					**References**
		**Fibro**	**Prolif**	**CD44**	**CD73**	**CD90**	**CD105**	**CD146**	**STRO-1**	***Nanog***	***Rex1***	***Oct4***	***Ki67***	**Osteo**	**Chondro**	**Adipo**	**Neuro**	**IPCs**	
DPSCs	Po	+	+	N/A	N/A	+	+	+	N/A	N/A	N/A	N/A	N/A	N/A	+	+	+	N/A	(104)
	Bo	+	+	N/A	N/A	+	N/A	N/A	N/A	N/A	N/A	N/A	N/A	N/A	N/A	N/A	N/A	N/A	(104)
	Ov	+	+	N/A	N/A	N/A	N/A	N/A	+	N/A	N/A	N/A	N/A	+	N/A	N/A	N/A	N/A	(73)
SED	Po	N/A	N/A	N/A	N/A	N/A	N/A	N/A	N/A	N/A	N/A	N/A	N/A	N/A	N/A	N/A	N/A	N/A	N/A
	Bo	N/A	N/A	N/A	N/A	N/A	N/A	N/A	N/A	N/A	N/A	N/A	N/A	N/A	N/A	N/A	N/A	N/A	N/A
	Ov	+	+	N/A	N/A	N/A	N/A	N/A	+	N/A	N/A	N/A	N/A	N/A	N/A	N/A	N/A	N/A	(73)
PDLSCs	Po	+	+	N/A	N/A	N/A	N/A	N/A	+	N/A	N/A	N/A	N/A	+	N/A	N/A	N/A	N/A	(105–108)
	Bo	N/A	N/A	N/A	N/A	N/A	N/A	N/A	N/A	N/A	N/A	N/A	N/A	N/A	N/A	N/A	N/A	N/A	N/A
	Ov	+	+	+	N/A	N/A	N/A	N/A	N/A	N/A	N/A	N/A	N/A	N/A	N/A	N/A	N/A	N/A	(109)
DFSCs	Po	+	+	+	N/A	+	+	N/A	N/A	N/A	N/A	N/A	N/A	+	N/A	N/A	N/A	N/A	(110, 111)
	Bo	+	+	N/A	N/A	N/A	N/A	N/A	N/A	N/A	N/A	N/A	N/A	+	N/A	N/A	N/A	N/A	(112)
	Ov	N/A	N/A	N/A	N/A	N/A	N/A	N/A	N/A	N/A	N/A	N/A	N/A	N/A	N/A	N/A	N/A	N/A	N/A
SCAP	Po	N/A	N/A	N/A	N/A	N/A	N/A	N/A	N/A	N/A	N/A	N/A	N/A	N/A	N/A	N/A	N/A	N/A	N/A
	Bo	N/A	N/A	N/A	N/A	N/A	N/A	N/A	N/A	N/A	N/A	N/A	N/A	N/A	N/A	N/A	N/A	N/A	N/A
	Ov	N/A	N/A	N/A	N/A	N/A	N/A	N/A	N/A	N/A	N/A	N/A	N/A	N/A	N/A	N/A	N/A	N/A	N/A
GMSCs	Po	N/A	N/A	N/A	N/A	N/A	N/A	N/A	N/A	N/A	N/A	N/A	N/A	N/A	N/A	N/A	N/A	N/A	N/A
	Bo	N/A	N/A	N/A	N/A	N/A	N/A	N/A	N/A	N/A	N/A	N/A	N/A	N/A	N/A	N/A	N/A	N/A	N/A
	Ov	N/A	N/A	N/A	N/A	N/A	N/A	N/A	N/A	N/A	N/A	N/A	N/A	N/A	N/A	N/A	N/A	N/A	N/A
BM-MSCs	Po	+	+	N/A	N/A	N/A	N/A	N/A	N/A	N/A	N/A	N/A	N/A	+	N/A	N/A	N/A	N/A	(110)
	Bo	+	+	N/A	N/A	N/A	N/A	N/A	N/A	N/A	N/A	N/A	N/A	+	N/A	+	N/A	N/A	(113)
	Ov	+	+	+	+	N/A	+	N/A	N/A	N/A	N/A	N/A	N/A	+	+	+	N/A	N/A	(114, 115)
AD-MSCs	Po	+	+	+	N/A	+	+	N/A	N/A	N/A	N/A	N/A	N/A	+	+	+	+	+	(116–124)
	Bo	+	+	+	+	N/A	N/A	N/A	N/A	N/A	N/A	N/A	N/A	+	N/A	+	N/A	N/A	(125)
	Ov	+	+	+	+	N/A	+	N/A	N/A	N/A	N/A	N/A	N/A	+	+	+	N/A	N/A	(126, 127)

DPSCs, dental pulp stem cells; SED, stem cells from exfoliated deciduous teeth; PDLSCs, periodontal ligament stem cells; DFSCs, dental follicle stem cells; SCAP, stem cells from apical papilla; GMSCs, gingival mesenchymal stem cells; BM-MSCs, bone marrow-derived mesenchymal stem cells; AD-MSCs, adipose-derived mesenchymal stem cells; Po, porcine; Bo, bovine; Ov, ovine; Fibro, fibroblast-like shaped; Prolif, Proliferative; Osteo, Osteogenic; Chondro, Chondrogenic; Adipo, Adipogenic; Neuro, neurogenic; IPCs, insulin-producing cells; +, positively expressed by proteins; +^G^, positively expressed by gene; N/A, not available.

**Table 3:** Potential cell source of oral and non-oral origin of laboratory animals.

**Source**	**Species**	**Characteristics**												**Potential differentiation**					**References**
		**Fibro**	**Prolif**	**CD44**	**CD73**	**CD90**	**CD105**	**CD146**	**STRO-1**	***Nanog***	***Rex1***	***Oct4***	***Ki67***	**Osteo**	**Chondro**	**Adipo**	**Neuro**	**IPCs**	
DPSCs	NHP (c)	+	+	+	+	+	+	N/A	N/A	+	+	+	N/A	+	+	+	N/A	N/A	(51)
	Rab	+	+	N/A	N/A	N/A	N/A	N/A	+	N/A	N/A	N/A	N/A	+	N/A	+	N/A	N/A	(128–130)
	Rat	+	+	N/A	N/A	N/A	+	N/A	N/A	N/A	N/A	N/A	N/A	N/A	N/A	N/A	N/A	N/A	(131)
	Mu	+	+	N/A	N/A	N/A	N/A	N/A	N/A	N/A	N/A	N/A	N/A	N/A	N/A	N/A	+	N/A	(132)
SED	NHP	N/A	N/A	N/A	N/A	N/A	N/A	N/A	N/A	N/A	N/A	N/A	N/A	N/A	N/A	N/A	N/A	N/A	N/A
	Rab	N/A	N/A	N/A	N/A	N/A	N/A	N/A	N/A	N/A	N/A	N/A	N/A	N/A	N/A	N/A	N/A	N/A	N/A
	Rat	N/A	N/A	N/A	N/A	N/A	N/A	N/A	N/A	N/A	N/A	N/A	N/A	N/A	N/A	N/A	N/A	N/A	N/A
	Mu	N/A	N/A	N/A	N/A	N/A	N/A	N/A	N/A	N/A	N/A	N/A	N/A	N/A	N/A	N/A	N/A	N/A	N/A
PDLSCs	NHP (b)	+	+	N/A	N/A	N/A	N/A	N/A	N/A	N/A	N/A	N/A	N/A	N/A	N/A	N/A	N/A	N/A	(133)
	Rab	+	+	N/A	N/A	N/A	N/A	N/A	N/A	+	N/A	+	N/A	+	+	+	N/A	N/A	(134)
	Rat	+	+	N/A	N/A	N/A	N/A	N/A	N/A	N/A	N/A	N/A	N/A	N/A	N/A	N/A	+	N/A	(135)
	Mu	+	+	N/A	N/A	N/A	N/A	N/A	N/A	N/A	N/A	N/A	N/A	+	N/A	N/A	N/A	N/A	(74)
DFSCs	NHP	N/A	N/A	N/A	N/A	N/A	N/A	N/A	N/A	N/A	N/A	N/A	N/A	N/A	N/A	N/A	N/A	N/A	N/A
	Rab	+	+	N/A	N/A	N/A	N/A	N/A	N/A	+	N/A	+	N/A	+	N/A	+	+	N/A	(134)
	Rat	+	+	+	N/A	+	N/A	N/A	N/A	N/A	N/A	N/A	N/A	+	N/A	+	N/A	N/A	(136, 137)
	Mu	+	+	+	N/A	N/A	+	N/A	N/A	N/A	N/A	N/A	N/A	+	N/A	N/A	N/A	N/A	(138)
SCAP	NHP	N/A	N/A	N/A	N/A	N/A	N/A	N/A	N/A	N/A	N/A	N/A	N/A	N/A	N/A	N/A	N/A	N/A	N/A
	Rab	N/A	N/A	N/A	N/A	N/A	N/A	N/A	N/A	N/A	N/A	N/A	N/A	N/A	N/A	N/A	N/A	N/A	N/A
	Rat	N/A	N/A	N/A	N/A	N/A	N/A	N/A	N/A	N/A	N/A	N/A	N/A	N/A	N/A	N/A	N/A	N/A	N/A
	Mu	N/A	N/A	N/A	N/A	N/A	N/A	N/A	N/A	N/A	N/A	N/A	N/A	N/A	N/A	N/A	N/A	N/A	N/A
GMSCs	NHP	N/A	N/A	N/A	N/A	N/A	N/A	N/A	N/A	N/A	N/A	N/A	N/A	N/A	N/A	N/A	N/A	N/A	N/A
	Rab	+	+	+	+	+	+	N/A	N/A	N/A	N/A	N/A	N/A	+	+	+	N/A	N/A	(139)
	Rat	+	+	+	+	+	+	N/A	N/A	N/A	N/A	N/A	N/A	+	+	+	+	N/A	(140–142)
	Mu	+	+	N/A	N/A	+	+	N/A	N/A	N/A	N/A	N/A	N/A	+	+	+	N/A	N/A	(141)
BM-MSCs	NHP (m)	+	+	N/A	N/A	+	+	N/A	N/A	+	+	+	N/A	+	+	+	N/A	N/A	(143)
	Rab	+	+	+	N/A	N/A	+	N/A	N/A	N/A	N/A	N/A	N/A	+	+	N/A	N/A	N/A	(144, 145)
	Rat	+	+	+	+	+	+	N/A	N/A	N/A	N/A	N/A	N/A	+	+	+	+	N/A	(51, 81)
	Mu	+	+	+	N/A	+	N/A	N/A	N/A	N/A	N/A	N/A	N/A	+	+	+	+	N/A	(81, 146, 147)
AD-MSCs	NHP (m)	+	+	N/A	N/A	N/A	N/A	N/A	N/A	N/A	N/A	N/A	N/A	+	+	N/A	N/A	N/A	(148)
	Rab	+	+	N/A	N/A	+	+	N/A	N/A	N/A	N/A	N/A	N/A	+	+	+	N/A	N/A	(149, 150)
	Rat	+	+	N/A	N/A	+	+	N/A	N/A	N/A	N/A	N/A	N/A	N/A	N/A	+	N/A	N/A	(151, 152)
	Mu	+	+	+	N/A	N/A	+	N/A	N/A	N/A	N/A	N/A	N/A	+	N/A	+	N/A	N/A	(153)

DPSCs, dental pulp stem cells; SED, stem cells from exfoliated deciduous teeth; PDLSCs, periodontal ligament stem cells; DFSCs, dental follicle stem cells; SCAP, stem cells from apical papilla; GMSCs, gingival mesenchymal stem cells; BM-MSCs, bone marrow-derived mesenchymal stem cells; AD-MSCs, adipose-derived mesenchymal stem cells; NHP, non-human primate; c, chimpanzee; b, baboon; m, macaque; Rab, rabbit; Mu, murine Fibro, fibroblast-like shaped; Prolif, Proliferative; Osteo, Osteogenic; Chondro, Chondrogenic; Adipo, Adipogenic; Neuro, neurogenic; IPCs, insulin-producing cells; +, positively expressed by proteins; N/A, not available.

## 4 Scaffolds and signaling molecules for OMF reconstruction

Scaffolds and signaling molecules have been comprehended as other essential components of tissue engineering to regenerate damaged tissues or organs (2). Scaffold is defined as a three-dimensional (3D) biomaterial for supporting the cells to proliferate and differentiate during the process of tissue regeneration (9). Furthermore, signaling molecules are crucial in the involvement of cellular responses (154). Thus, besides stem cells, scaffolds and signaling molecules are suggested to have the potential to be used for OMF reconstruction (155).

### 4.1 Scaffolds for OMF tissue regeneration

Selecting an appropriate scaffold for each specific tissue is an essential factor in tissue engineering. The biomaterial and scaffold are a transport device for cells and signaling molecules to promote tissue regeneration (9, 156, 157). Moreover, the scaffold structure functions as a reservoir for water, nutrients, cytokines, and growth/differentiation factors (27). Therefore, scaffold fabrication should be considered for its functionality, biocompatibility, biodegradability, mechanical properties, and structure characteristics such as pore size, porosity, and interconnectivity (158, 159). Addition, the 3D structural scaffold can be printed to resemble donor tissue architecture (9). Also, the designated material is expected to support cell attachment, migration, proliferation, differentiation, maturation, and ECM production (19, 160).

Scaffold materials can be broadly categorized into natural, and synthetic (157). Biomaterials can be classified into metal, ceramic, polymer, and composite (161). The advantages and disadvantages of each material should be taken into consideration before utilizing them for OMF hard or soft tissue regeneration and based on the desired functionality of the material on a specific tissue.

As for scaffold-free approaches, techniques include self-organization (cell sheet and aggregate engineering) and self-assembly processes. Interestingly, scaffold-free approaches can be applied for OMF regeneration, especially for the indication of skin, TMJ disc, cartilage, bone, and periodontal ligament tissues (7, 8, 162).

#### 4.1.1 Biomaterials and scaffold characteristics for OMF complex tissue regeneration

Titanium alloy prostheses have been of interest for OMF hard tissue repair applications (163). However, this material often leads to local tissue damage and chronic inflammatory response due to poor biomechanical properties and low biocompatibility (164) and other materials that may exhibit superior qualities as well as practical advantages and disadvantages are available. In this regard, two important properties to consider are the osteoinductive and osteoconductive potential of the chosen scaffold material especially when applied for bone regeneration purpose (83).

Other materials that can be used for bone regeneration include ceramics, synthetic polymers, and natural polymers. Natural polymer-based scaffolds are known to be biologically active and able to enhance cell adhesion and growth. Despite these properties, limitations in fabrication and inferior mechanical characteristics represent mechanical property constraints compared with other scaffold types, resulting in major disadvantages of this type of scaffold for bone regeneration (157, 165, 166). One study reported starch as natural polymer could not be used directly due to its poor stability and mechanical properties. Improvement of mechanical properties was gained by reinforcement with HA as composite (167). Examples of natural polymers employed for bone tissue regeneration include collagen, gelatin, chitosan, alginate, silk proteins, hyaluronic acid, fibrin, and keratin (168). In contrast, synthetic polymers exhibit elastic properties, endless forms, established structures, and comparable characteristics to biological tissues that are predictable and reproducible (169). However, the main disadvantages of synthetic biomaterials are lack of cell adhesion sites, the need to chemically modify them to improve cell adhesion, and some materials produce toxic by-products for surrounding cells and tissues (157, 169). Biodegradable synthetic materials that are popular for bone tissue engineering include polylactic acid (170), polyglycolic acid (PGA), polycaprolactone (PCL), poly(lactic-co-glycolic) acid (PLGA), and polyurethane (PU) (169). Lastly, ceramics are biocompatible and suitable for replacing hard tissues, but their brittleness represents a drawback. Common ceramic scaffolds used for bone regeneration are HA and TCP (171). Some of the disadvantages of ceramic scaffolds can be minimized by fusing one specific type of material with another to generate a hybrid with enhanced regenerative potential (172).

#### 4.1.2 Biomaterials and scaffold characteristics for OMF soft tissue regeneration

Oral and maxillofacial soft tissue engineering is meant to reconstruct lip, skin, salivary gland, oral mucosa, muscle, ligament, and TMJ-related tissues, or periodontal tissues. Soft tissue engineering facilitates defect reconstruction for soft tissues (173). Unlike hard tissue, scaffold materials for soft tissues vary in their flexibility (9). Since vascularization has been the central issue of soft tissue engineering, a strong blood supply must be achieved for successful soft tissue reconstruction (173). Thus, scaffolds are required to be able to carry blood supply via vascular ingrowth. Additionally, engineered scaffolds should be adjusted to shape and size of the defect and accommodate the appropriate cells, thus sensory and motoric functions of the damaged tissues would be regained (173).

Several materials have been reported for use in OMF soft tissue engineering. Hydrogels (HG) play a prominent role given that they can be generated from various materials, such as chitosan, chitin, hyaluronic acid, gelatin, peptide, PLGA, and PEG. One study mentioned that HG is preferable for soft tissue but not hard tissue reconstruction because its mechanical properties do not support load bearing (174). Despite that, a recent review noted that HG can regenerate periodontal soft and hard tissue as well as dental pulp, dentine, and enamel (175). Another study showed that commercial biodegradable scaffolds made of either a combination of collagen fibers porous matrix and glycosaminoglycan, polyglycin 910, or collagen membrane and collagen I, can generate an oral mucosa equivalent (OME) *in vitro* (176). Moreover, a silk fibroin/hyaluronic acid-based scaffold has been suggested for soft tissue regeneration (177). Silk fibroin has also been combined with gelatin and chitosan into a mimicked scaffold *in vitro* using human keratinocyte cells for OMF soft tissue engineering applications (178). In addition, a previous study explored the potential of polyglycolic acid polymer scaffolds for salivary gland tissue engineering (179). To be used in the veterinary medicine field, such materials should meet general consideration from material selection, design and manufacturing, design control and testing, to safety and efficacy before going to the clinical studies to meet the product purpose (180).

In this review, we have included cartilage as a soft tissue pertinent to the OMF region, specifically at the TMJ. Like scaffolds in general, the fabrication of a scaffold for cartilage should take into consideration its composition and biological properties, its architecture, and its mechanical and degradation (181). Materials that have been studied for TMJ regeneration, which are intended to mimic both cartilage and bone tissues, include PLA disc (182), PGA (183), chitosan/alginate (184), fibrin/chitosan (185), biphasic scaffolds of HA/TCP and hyaluronic acid (170), and PGA and PLGA/polyethylene glycol (PEG) (186).

### 4.2 Signaling molecules for OMF tissue regeneration

Signaling molecules represent a crucial aspect when developing strategies for OMF tissue engineering. These molecules refer to growth factors, which are proteins produced by the cells (27). Current technologies allow the production of growth factors using recombinant methods. By binding to their receptors, signaling molecules activate intracellular signaling events that can trigger or inhibit cell adhesion, proliferation, migration, and differentiation. Thus, this approach gives rise to regenerating damaged tissue. Moreover, signaling molecules can be applied as a single treatment and adsorbed on a scaffold, as previously mentioned above. This combination enhances the integration between the material and host tissues (27).

Many signaling molecules have been investigated for their therapeutical potential in context of OMF regeneration, and even, some are clinically approved by the Food and Drug Administration (187). Several well-known signaling molecules include bone morphogenetic proteins (BMPs), transforming growth factor-β (TGF-β), platelet-derived growth factor (PDGF), fibroblast growth factor (FGF), vascular endothelial growth factor (VEGF), and insulin-like growth factor (IGF) (188). Among these, BMPs are the most widely studied for bone regeneration, and several types of BMPs have been mentioned to have crucial roles in bone morphogenesis and bone defect repair and regeneration (189, 190). Interestingly, rhBMP-2 and rhBMP-7 have been approved by the Food and Drug Administration (187) for bone regeneration applications (189, 190). In general, BMPs are considered very relevant for OMF regeneration (191), being capable of osteoinductive on MSCs (190). On the other hand, TGF-β has also been proposed as a potent modulator of bone regeneration with the ability to enhancing osteogenesis at a low concentration (192). Paradoxically, TGF-β at a high concentration inhibits osteogenesis. Another molecule called teriparatide has also been of interest to OMF surgeons. Teriparatide is a recombinant parathyroid hormone used to treat osteoporosis (193). Moreover, teriparatide is approved by the FDA for treatment of osteoporosis and has been reported to regenerate jawbone defects (194). Finally, the potential of leptin to enhance oral mucosa regeneration by increasing vascularization has been reported (195).

## 5 Translation to clinical practice

Animal experiment models have been introduced to study the translational treatment of OMF tissue engineering in clinical practice by mimicking real clinical situations. In this review, we provided most of the *in vivo* studies employing non-human MSCs and successful treatment in veterinary patients with OMF defects. The data are expected to be used for further translation to treat OMF defects in veterinary patients.

### 5.1 Oral hard tissue engineering in an animal model

Periodontal tissue regeneration in periodontitis-induced beagles has been reported to using rhBMP-2 adsorbed on PLGA-gelatin sponge carrier material treatment (196). Another report demonstrated that autologous canine DPSCs combined with a commercial xenograft scaffold in a periodontitis-induced canine model had the ability to achieve periodontal tissue regeneration after 8 weeks of transplantation (197).

Another published study performed mandibular osteotomy in beagles to create a bone defect and treated it with a 3D-printed HA scaffold co-cultured with canine BM-MSCs (164). They found that the seeded cells played an essential role in large bone defects by differentiating into osteoblasts, regulating immune response, and providing a microenvironment for tissue regeneration. Also, De kok et al. reported that canine BM-MSCs enhanced bone formation when combined with a HA/TCP scaffold in an alveolar defect canine model (198). In addition, SHEDs have been transplanted with a scaffold in a mandibular defect canine model (199). However, this study showed no significant difference in bone regeneration compared with the control group. Nevertheless, the authors demonstrated no tumorigenesis or severe inflammation after transplantation (182, 200).

Evaluation of maxillary sinus augmentation (MSA) has also been performed in canine by reconstructing it with autologous osteoblasts on a β-TCP scaffold (201). It was shown that the addition of osteoblasts to the scaffold improved maxillary regeneration by the remarkable differences of height and volume of augmented maxillary sinus compared to groups with scaffold only and autogenous bone graft (201).

Allogenic transplantation for pulp regeneration study has been reported using the canine model. After pulpectomy, canine DPSCs transplantation was conducted in dogs with consideration given matched and mismatched dog leukocyte antigen (DLA). The authors revealed that the both DLA matched and mismatched allogenic transplantation are safe and effective for pulp regeneration due to the lack of signs for toxicity and the generation of pulp-like tissues 12 weeks post-transplantation (202). The results are supported by another study revealing canine DPSCs do not express HLA-DR (similar to DLA Class II in dogs) by flow cytometry (49). In addition, one study explored the blood type- and breed-associated immune reaction on allogenic transplantation in equines. The authors revealed that universal blood donor-type of Standardbred has been suggested to be less likely occurred in a study of equine BM-MSCs with low expression of MHC Class II (similar to DLA Class II in dogs) than non-blood donor type and in Thoroughbred. This study implies that breed of donor should be considered with the use of universal blood donor-type (203).

### 5.2 Oral soft tissue engineering in an animal model

Regarding soft tissue, Qian et al. revealed that combining leptin, silk fibrin, and polydopamine improves oral mucosa healing and triggers blood vessel regeneration in New Zealand rabbits (195). In addition, regeneration of oral mucosa has been previously simulated in nude mice with skin defects implanted with a human gingival endothelial cells (HGECs)-human gingival fibroblasts (HGFs)-vascular endothelial cells (VECs)-acellular vascular matrix (ACVM)-0.25% human-like collagen I (HLC-I) complex (204). In such study, investigators found that the scaffold complex had a regenerative effect based on the presence of epithelioid-like, lamina propria-like, and vascular-like structures observed on histopathological analysis.

As for the salivary gland, one study showed that 3D hyaluronic acid-based hydrogel scaffold implantation in rats was able to adhere around the parotid tissue (205). Interestingly, the implanted scaffold seeded with human salivary cells was able to retain its spheroid structure and express CD44 and receptor for hyaluronan-mediated motility (RHAMM/CD168; a progenitor cell marker). Another study has shown that human salivary cells seeded on unwoven sheets of polyglycolic acid polymers are able to form functional tissues in mice (179). These two studies indicate that both scaffolds have potential for salivary gland regeneration.

*In vivo* TMJ studies have been reported. Replacement of TMJ disc in New Zealand rabbits with PLA discs containing autologous AD-MSCs was demonstrated (182). The authors presented that the material has the potential for further use yet needs more dissemination. In addition, a porcine urinary bladder matrix (UBM) scaffold was implanted in the TMJ in a dog model (200). They found that the material was inductive and suggested that an extracellular matrix (ECM)-based scaffold can be a solution for TMJ disc replacement. In addition, a study of scaffold-free TMJ implant from costal chondrocytes has been reported in the minipigs as TMJ disc regeneration model. The study found that the implants are safe, able to prevent degeneration of disc thinning, and even, regenerate the defects by refining the osteoarthritis score for the efficacy (162).

Despite the positive outcomes of the cell-based treatment, there has been reports that MSCs provide no clinical improvement in clinical cases. Autologous bone marrow aspirate concentrate, adipose stromal vascular fraction, and allogeneic human umbilical cord tissue-derived MSCs (UC-MSCs) showed no significant difference on beneficial outcomes for osteoarthritis in a phase 3 trial comparing with conventional therapy, corticosteroid injection (CSI) (206). A meta-analysis report also showed that stem cell infusion did not result in clinical improvement for acute myocardial infarction (207). According to the reports, such strategies could be made starting from identifying the possibilities of non-beneficial outcomes, re-designing the experiment, and evaluating the new strategy.

### 5.3 Oral and maxillofacial tissue engineering in a clinical case

Some of the previously mentioned *in vivo* studies and corresponding results offer the opportunity to leverage tissue engineering solutions to repair OMF defects in veterinary patients. Tissue engineering is a multidisciplinary field consisting of medicine, material science, engineering, and biology. The prospective tissue-engineered products could be either comprising of cells, signaling molecules, scaffolds, or combination of scaffold with cells, or signaling molecules (208). Thus, according to the medical product classification by FDA, the products are either biological, medical device, or combination of biological and medical device. In addition, to prove the safety and efficacy in the clinical case, such studies should be performed as well. Indeed, several case studies have been reported in the veterinary literature illustrating broad potential for clinical use (209, 210).

In one case reported by Spector and Boudrieau (210), a partial mandibulectomy performed in a cocker spaniel to treat an odontoma resulted in a large 5-cm defect impacted functionality and quality of life. The defect was reconstructed using rhBMP-2 delivered in an absorbable collagen sponge containing HA/TCP granules (compression resistant matric [CRM]) and plate fixation. The results demonstrated that the correction was successful without any significant complications, either in functional or cosmetic aspects, after 36 months. The authors also suggested that rhBMP-2 can be an alternative to reconstructive techniques for dog use, increasing cost efficiency by using a lower dose than in humans. This initial methodology was further described and expanded on. Case series of segmental mandibulectomies and bilateral rostral mandibular reconstruction and chronic, defect union fracture treatment have been reported to be resolved by the use of plate and CRM infused with rhBMP-2 (211–213). Additionally, Tsugawa et al. reported a retrospective study of the promising mentioned methodology for canine acanthomatous ameloblastoma (CAA) (214). The studies showed that margin between implant material and native bone become indistinct at week 4 or later post-operatively without relatively minimum complications. Similarly, autologous and allogeneic mesenchymal stem cells derived from adipose tissue were reported for clinical use in a sample of cats with refractory feline chronic gingivostomatitis (215, 216). These studies suggest that both cell sources are relatively safe and potentially effective even though treatments were not compared to other types of intervention.

An interesting clinical application of a 3D-printed PCL/β-TCP scaffold has also been reported in a maxillary bone defect due to an oral squamous cell carcinoma in a 12-year-old female mixed breed dog (209). Reconstruction was successful based on computed tomography images 2 months after surgery.

Several animal studies were presented with beneficial outcomes of investigated technologies. However, some products are not intended for veterinary patients (179, 195, 196, 199–201, 204, 205). Given the high success rates of OMF tissue engineering reported in experimental animal models and selected clinical cases, tissue engineering for OMF defect repair appears to be a viable alternative in veterinary medicine. To a greater extent, proposed and safety- and efficacy-proven OMF tissue engineering products can be registered for an intellectual property (IP) for veterinary patients.

## 6 The future challenge for OMF tissue engineering in veterinary medicine

**Figure 2:**
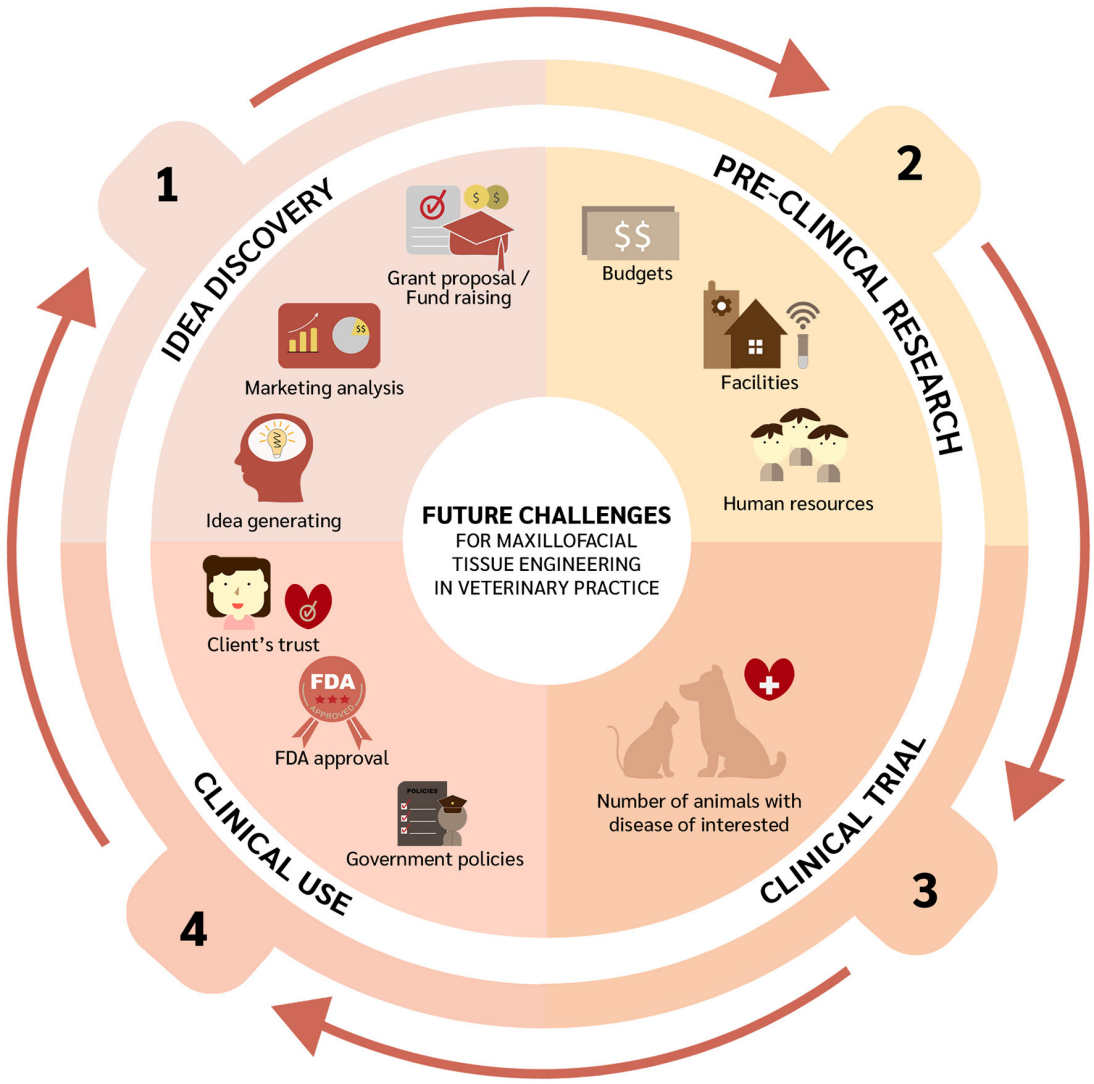
Future challenges of oral and maxillofacial tissue engineering in veterinary medicine.

Despite the immense potential of tissue engineering for OMF defect repair in veterinary medicine, several milestones and challenges lie ahead (Figure 2). Firstly, generating ideas by gathering information on clinically relevant OMF diseases, identifying proper funding mechanisms to conduct pertinent bench and clinical research, and conducting market analysis to explore translational feasibility. Similarly, the potential of stem cells from one specific source and species origin should be further investigated. Indeed, cells need to be more exhaustively characterized. This can be following from the proposed mechanisms of MSCs in differentiation to the targeted tissue, immunomodulation property, and as endocrine secretors (217, 218). Such characterization and *in vivo* experiments are essential because they will determine cell potency. *In vivo* studies related to OMF surgery can be performed following the treatment interest since one type of cell may have different potential with another cell in terms of kind of treatment. The topic has been raised by a previous study (219) and also applies to scaffolds and signaling molecules. In addition, tissue engineering as personalized medicine should be considered as well with the 3D printing technologies to adapt with the patients' needs (220). Research on the immune response after transplantation using adequate animal models is also required. Rejection of a transplant may occur due to several factors, such as the source of the transplant (i.e., auto-, allo-, or xenogeneic), dose, and route of administration. Moreover, any pre-clinical experiments need to be performed considering any human resources, facilities, and budget available. Before implementing novel therapies, properly designed clinical trials should be conducted. Since clinical studies require blinded and randomized interventions, sample sizes must be met to ensure robust and reproducible results, which is often a challenge in veterinary medicine. Moreover, any eventual translation of interventions into clinical practice would require building up clients' trust and very likely third-party involvement. Finally, Ivanoska et al. have noted logistical challenges including product transport and cell delivery (219).

## 7 Conclusion

Based on the experimental and clinical studies, regenerative tissue engineering is a promising approach with immense veterinary translational potential for the reconstruction of OMF defects in animals. However, the types of tissue engineering components should be chosen appropriately based on past, current, and future research in order to achieve optimal outcomes based on tissue regeneration.

## Author contributions

SDP: Writing—original draft. TT: Writing—original draft. MP: Writing—original draft. TH: Writing—original draft. SP: Writing—review & editing. NF: Writing—review & editing. CS: Writing—review & editing. SR: Writing—review & editing.
